# Good-eating-quality QTLs detected in two breeding populations by genome-wide association mapping increase eating quality of the Japanese rice cultivar ‘Koshihikari’

**DOI:** 10.1270/jsbbs.25025

**Published:** 2025-10-24

**Authors:** Yoshinobu Takeuchi, Toshio Yamamoto, Jun-ichi Yonemaru, Yoko Takemoto-Kuno, Shuichi Fukuoka, Makoto Kuroki, Akitoshi Goto, Kazuki Matsubara, Hiroyuki Sato, Hideyuki Hirabayashi, Nobuya Kobayashi, Masayuki Yamaguchi, Takuro Ishii, Ikuo Ando

**Affiliations:** 1 Institute of Crop Science, National Agricultural and Food Research Organization (NARO), 2-1-2 Kannondai, Tsukuba, Ibaraki 305-8518, Japan

**Keywords:** *Oryza sativa* L., eating quality, quantitative trait locus, genome-wide association mapping, chromosome segment substitution line

## Abstract

To identify QTLs controlling the eating quality of ‘Akidawara’, ‘Satojiman’, and ‘Ikuhikari’ rice, we performed a genome-wide association mapping analysis using two breeding populations in 2013 and 2014 derived from crosses between these and another parental line. Through sensory tests by a trained panel, we evaluated five components of the eating quality of cooked rice. Fifty-eight QTLs for these components were detected in breeding lines in 2013 (seven regions of chromosome [chr.] 1, 4, and 11) and 2014 (ten of chr. 1, 2, 4, 8, 9, and 11). The Akidawara, Satojiman, or Ikuhikari alleles at these QTLs increased eating quality. QTLs on the short arm of chr. 4, the middle of the long arm of chr. 4, the distal end of the long arm of chr. 4, and the short arm of chr. 11, were identified in both years. The genetic effects of the Satojiman alleles at QTLs on the distal end of the long arm of chr. 4 and on the short arm of chr. 11 were confirmed by analysis of two chromosome segment substitution lines containing a Satojiman segment in the ‘Koshihikari’ background in 2016 and 2017, in which the Satojiman alleles increased the level of eating quality of Koshihikari.

## Introduction

The *japonica* rice cultivars, ‘Akidawara’, ‘Satojiman’, and ‘Ikuhikari’, are grown over a wide area in Japan and are favored by Japanese consumers because of their good eating quality (Ando *et al.* 2011, Sato *et al.* 2013, Tomita *et al.* 2005). Eating quality of Akidawara, Satojiman, and Ikuhikari, was different with that of ‘Koshihikari’. Akidawara and Satojiman have good hardness, stickiness, and taste. Ikuhikari has good glossiness and hardness. In addition, Akidawara, Satojiman, and Ikuhikari have good agronomic characteristics, including high grain weight (Ando *et al.* 2011). Therefore, they are used as parental cultivars to develop new rice cultivars aiming to the introduction its excellent good characteristics, such as eating quality and grain weight.

Eating quality is an important trait in rice breeding. In general, it is evaluated through sensory testing by trained panelists. Although its reliability depends on the experience of the panelists, the sensory test is recognized as the most effective method to determine eating quality, and is used extensively for selection in rice breeding. The eating quality of cooked rice is evaluated from several components, notably glossiness (GL), hardness (HA), stickiness (ST), taste (TA), and overall evaluation (OE) (Bett-Garber *et al.* 2001, Yamamoto and Ogawa 1992). As this evaluation must be done on advanced generations of breeding materials because of the requirement for large amounts of grain, it is very time consuming and labor intensive. Studies designed to develop a more efficient system for screening eating quality have revealed that amylose content (AC) and protein content (PC) of the endosperm are strong determinants of eating quality (Ishima *et al.* 1974, Juliano *et al.* 1965, Juliano 1985), but do not fully explain it (Bett-Garber *et al.* 2001). Thus, other genetic factors remain to be uncovered.

Recently, marker-assisted selection (MAS) has been used to develop new cultivars with particular traits (Yamamoto and Yano 2008). It has been used to introduce bacterial blight resistance, blast resistance, lodging resistance, high yield potential, high adaptability, and submergence tolerance (Ashikari *et al.* 2005, Hayashi *et al.* 2004, Neeraja *et al.* 2007, Singh *et al.* 2001, Sugiura *et al.* 2004, Takeuchi *et al.* 2006, Wang *et al.* 2005). Its effectiveness depends on the reliability of markers linked to the target gene loci. For example, high-resolution mapping and the use of tightly linked DNA markers have allowed the development of isogenic lines of Koshihikari with either early or late heading controlled by a very tiny chromosome segment (150–625 kb) (Takeuchi *et al.* 2006). In terms of MAS for eating quality, only the *Waxy* (*Wx*) gene has been manipulated so far (Sato *et al.* 2002, Suzuki *et al.* 2003).

Several genetic analyses of eating quality have been conducted (Takeuchi *et al.* 2007, 2008, Tanaka *et al.* 2006, Wan *et al.* 2004). Genetics analyses have also been performed to detect quantitative trait loci (QTLs) for eating quality by using crosses between distantly related *japonica* and *indica* cultivars (Takeuchi *et al.* 2007, Wan *et al.* 2004). Those analyses detected a major QTL in the region of the *Wx* gene on the short arm of chromosome [chr.] 6, suggesting that the difference in AC was due to the allelic difference at *Wx* between *japonica* (*Wx^b^*) and *indica* (*Wx^a^*) cultivars.

As such studies might not be able to identify genetic factors unrelated to AC controlling eating quality, it is still necessary to develop mapping populations derived from crosses between a *japonica* and other *japonica* cultivars with *Wx^b^*. Takeuchi *et al.* (2008) identified several QTLs for eating quality in two kinds of backcross inbred lines (BILs) derived from a cross of a *japonica* cultivar ‘Nipponbare’/Koshihikari//Nipponbare and a cross of Nipponbare/Koshihikari//Koshihikari. One of those QTLs explained 11.6% of the total phenotypic variance. The major QTL at the distal end of the short arm of chr. 3 was commonly identified in both BILs. The Koshihikari alleles at the QTL increased eating quality. The eating quality due to the Koshihikari alleles at the QTL on the short arm of chr. 3 not related to the AC. Also, Ando *et al.* (2011) reported that *japonica* cultivars such Akidawara and Ikuhikari have the Koshihikari alleles at the eating quality QTL on the distal end of the short arm of chr. 3. However, the other genetic basis of the eating quality of Akidawara, Satojiman, and Ikuhikari is few understood. To apply MAS to eating quality, it is necessary to identify other QTLs in these cultivars.

QTL mapping has revealed the genetic architecture of complex agronomic traits in rice (Yamamoto *et al.* 2009). However, genetic diversity in rice breeding materials in Japan is small. On account of the limited genome resolution, the detection of QTLs for phenotypic variations in breeding materials has been difficult. Recently, the single nucleotide polymorphism (SNP) analysis has allowed the elucidation of detailed information on genetic polymorphisms in breeding materials (Yamamoto *et al.* 2010). The advent has enabled the detection of QTLs in breeding materials with small genetic variations by using nonparametric analysis (Kruglyak and Lander 1995). In this study, we report the detection of QTLs for eating quality in breeding materials derived from crosses between Akidawara, Satojiman, or Ikuhikari and other lines. We identified new QTLs on the distal end of the long arm of chr. 4 and the short arm of chr. 11 controlling the eating quality of Satojiman, respectively. The genetic effect of the Satojiman alleles at these QTLs was confirmed by the development of two chromosome segment substitution lines (CSSLs) and a phenotype assay.

## Materials and Methods

### Plant materials

We used two breeding populations—68 lines in 2013 and 91 lines in 2014—derived from crosses between other lines and *japonica* cultivar Akidawara, Satojiman, or Ikuhikari (Supplemental Table 1). The 68 and 91 lines, and their parents were raised at the National Institute of Crop Science (Yawara, Tsukubamirai, Ibaraki, Japan). Seeds were sown on 2 May and seedlings were transplanted on 27 to 30 May in their years. In their years, the planting density was 22.2 plants m^–2^ (two-row plots, 15 cm × 30 cm). Nitrogen, phosphorus, and potassium fertilizers were each applied at 8 g m^–2^ in each year. Sixty-four plants per line were raised with two replications in each year. Sixty out of the 64 plants per line were harvested at maturity. All the seeds were air-dried in a greenhouse, threshed, and hulled. Fully matured grains were used for evaluation of eating quality.

To verify the allelic effects of the QTLs detected, we selected two plants, NG-5 and NG-11, from advanced backcross progeny (BC_3_F_2_, 224 plants) on the basis of the genotypes of SNP markers that showed homozygous for the Satojiman alleles on a segment of the distal end of the long arm of chr. 4 and the short arm of chr. 11 in a homozygous Koshihikari background, respectively. NG-5, NG-11, Koshihikari, and Satojiman were grown in a paddy field at the National Institute of Crop Science (Yawara, Tsukubamirai, Ibaraki, Japan), in 2016 and 2017. Seeds were sown on 2 May and seedlings were transplanted at 23.1 plants m^–2^ on 30 May in their years. Fertilizer was applied as described above. Thirty plants with two replications were harvested at maturity. All the seeds were air-dried in a greenhouse, threshed, and hulled. Fully matured grains were used for evaluation of eating quality and chemical properties.

### Sensory test of eating quality

The sensory test was performed according to the method of Yamamoto *et al.* (1996). Five hundred grams of hulled grain was polished to a yield of ~90% in a rice mill (VP-31T; Yamamoto Co. Ltd., Yamagata, Japan). Then 350 g of polished rice was placed in the bowl of a rice cooker (MB-YH16; Mitsubishi Electric Co. Ltd., Tokyo, Japan) and washed five times with water. The washed rice was soaked in water for 30 min, and cooked for about 30 min at a 1.4:1 (w/w) ratio of water to polished rice. The cooked rice was then steamed in the cooker for an additional 10 min. The rice was evaluated by a panel of 20 judges (9 men and 11 women, ages 27 to 48 years), who had been trained for over 2 years in the scoring of each component of eating quality. In fact, the panel had been achieved the selections of new cultivars such as Akidawara and Satojiman with good eating quality by the sensory test. Nine lines were evaluated in the one sitting. Filtered water was used to cleanse the mouth before testing each sample. The judges evaluated GL, TA, ST, HA, and OE. GL was scored by the degree of glossiness of the surface of the cooked rice. TA was scored by the degree of sweetness or bitterness. ST was scored by the degree of the force required to remove the cooked grains from upper teeth and lower teeth. HA was scored by the degree of the force required to compress the cooked grains between the upper and lower teeth. OE score was determined from the total scores and balance of GL, TA, ST, and HA. The GL, TA, ST, and OE scores of Nipponbare were rated –1 (slightly low) relative to Koshihikari, and the HA score of Nipponbare was rated +1 (slightly hard). A scale between Nipponbare and Koshihikari was used to determine the score of each component of eating quality of each line. The eating quality of each line was given scores from –5 (extremely poor) to +5 (excellent), compared with that of the reference cultivar Koshihikari (score = 0). The scores from the 20 judges were averaged.

### Analysis of amylose and protein contents

We crushed polished rice in a cyclone mill (SFC-S1; UDY Corp., CO, USA). The resulting flour was diluted with 0.5 *N* NaOH and left overnight at room temperature. After dilution to 0.05 *N* NaOH with water, AC was determined by colorimetry with iodine (Juliano 1971). PC was measured by the Bradford assay (XL-Bradford; APRO Science Corp., Tokushima, Japan). AC and PC of each line were determined in three different samples. The average AC and PC values were used for statistical analysis.

### Analysis of SNP markers

We determined the genotypes of breeding lines in 2013 and 2014, and of the two CSSLs, NG-5 and NG-11 in 2016 at 1297 SNP markers covering all 12 chromosomes (Nagasaki *et al.* 2010, Yamamoto *et al.* 2010). We selected the 1297 SNPs with array-signals in the Akidawara, Satojiman, and Ikuhikari genomes, out of 1917 designed SNPs with array-signals in Koshihikari and Nipponbare genomes. Total DNA was extracted from a small piece of leaf of each line. The DNA was used for genotyping by the GoldenGate Bead Array Technology Platform and the BeadStation 500G system (Illumina, CA, USA).

### Genome-wide association QTL mapping

We performed QTL analysis of eating quality by using genotype data of the 1297 SNP markers. Putative QTLs in the breeding lines were detected by a nonparametric test using Jonckheere-Terpstra test (Kruglyak and Lander 1995). Owing to the nature of sensory testing, we used a stringent threshold (*P* = 0.001) to declare a putative QTL.

## Results

### Phenotypic variations in 68 lines in 2013 and 91 lines in 2014

**Fig. 1.**
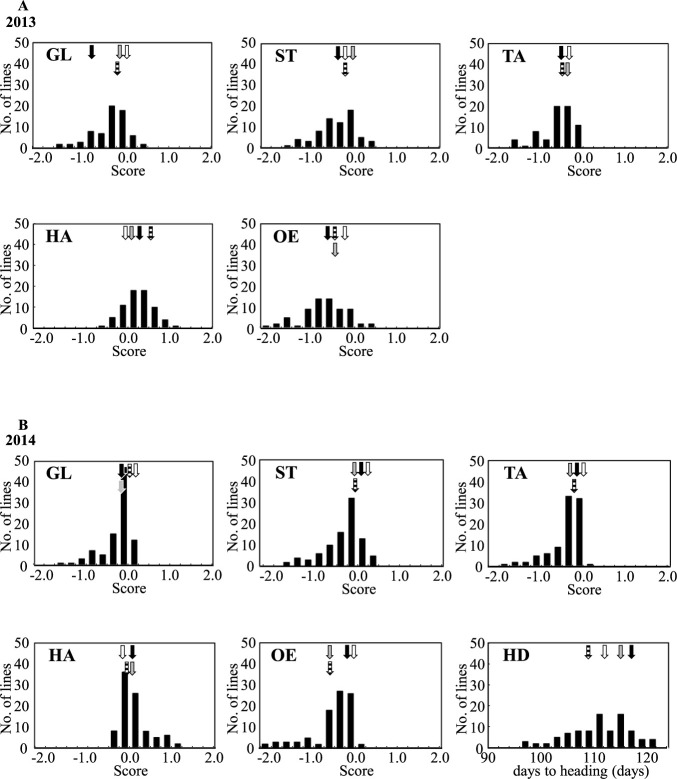
Frequency distribution of glossiness (GL), taste (TA), stickiness (ST), hardness (HA), overall evaluation (OE), and days to heading (HD) in 68 lines in 2013 (A) and 91 lines in 2014 (B). *Gray*, *black*, *stripe*, and *white* arrows indicate the mean values for Akidawara, Satojiman, Ikuhikari, and Koshihikari, respectively.

In 2013, the mean scores of the five components of eating quality of four parental cultivars, Akidawara, Satojiman, Ikuhikari, and Koshihikari, were –0.14, –0.83, –0.17, and –0.08 for GL, –0.43, –0.58, –0.50, and –0.33 for TA, –0.14, –0.42, –0.25, and –0.25 for ST, 0.07, 0.25, 0.50, and –0.08 for HA, and –0.50, –0.67, –0.50, and –0.25 for OE, respectively (Fig. 1A). In 2014, these score of five components of Akidawara, Satojiman, Ikuhikari, and Koshihikari were –0.13, –0.14, 0.00, and 0.14 for GL, –0.33, –0.21, –0.25, and –0.14 for TA, –0.20, 0.07, –0.19, and 0.21 for ST, 0.07, 0.07, –0.13, and –0.21 for HA, and –0.40, –0.14, –0.31, and –0.07 for OE, respectively (Fig. 1B). The days to heading of Akidawara, Satojiman, Ikuhikari, and Koshihikari were 115, 117, 109, and 112 in 2014. All traits in both lines showed continuous variations beyond the range of parental values (Fig. 1).

### QTLs detected in 68 lines in 2013

**Fig. 2.**
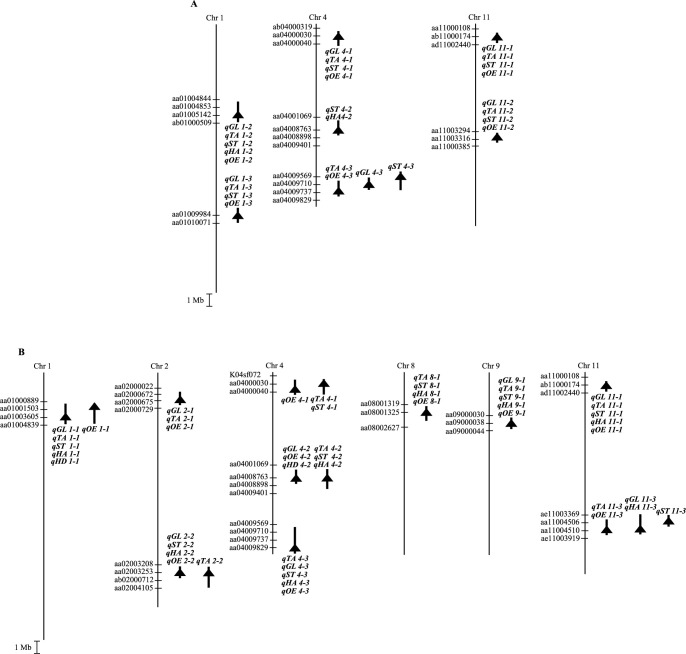
Putative QTLs for eating quality detected in 68 lines in 2013 (A) and 91 lines in 2014 (B). The chromosome number is shown at the *top*. *Vertical bars* denote the physical map. Putative QTLs for eating quality were detected by GWAS analysis. *Bold vertical bars* indicate the most likely chromosomal regions for the putative QTL within a certain confidence interval (defined by a decrease of 0.5 from the peak LOD values). Triangle indicates the nearest marker locus revealed by GWAS analysis. *Black* triangle indicates that the Akidawara, Satojiman, or Ikuhikari alleles increase the trait score. Abbreviations are as follows: GL, glossiness; TA, taste; ST, stickiness; HA, hardness; OE, overall evaluation; HD, days to heading. The prefix “NIAS_Os_” has been omitted from the marker names for brevity.

We detected 27 QTLs for eating quality in the 68 lines (Table1, Fig. 2A). Five QTLs (*qGL1-2*, *qTA1-2*, *qST1-2*, *qHA1-2*, and *qOE1-2*) were detected on the centromere region of chr. 1 (near SNP marker NIAS_Os_aa01005142). Four QTLs (*qGL4-1*, *qTA4-1*, *qST4-1*, and *qOE4-1*) were detected on the short arm of chr. 4 (near NIAS_Os_aa04000030). Four QTLs (*qGL4-3*, *qTA4-3*, *qST4-3*, and *qOE4-3*) were detected on the distal end of the long arm of chr. 4 (near NIAS_Os_aa04009569, NIAS_Os_aa04009710, and NIAS_Os_aa04009737). Four QTLs (*qGL11-2*, *qTA11-2*, *qST11-2*, and *qOE11-2*) were detected on the centromere region of chr. 11 (near NIAS_Os_aa11003316). The Satojiman alleles at all QTLs improved the eating quality. In addition, four QTLs (*qGL1-3*, *qTA1-3*, *qST1-3*, and *qOE1-3*) were detected on the long arm of chr. 1 (near SNP marker NIAS_Os_aa01009984). The Akidawara and Ikuhikari at these QTLs improved the eating quality. Two additional QTLs, *qST4-2* and *qHA4-2*, were detected on the middle of the long arm of chr. 4 (near SNP marker NIAS_Os_aa04008763). The Akidawara, Satojiman, and Ikuhikari alleles at two QTLs improved the eating quality. Furthermore, four QTLs (*qGL11-1*, *qTA11-1*, *qST11-1*, and *qOE11-1*) were detected on the short arm of chr. 11 (near NIAS_Os_ab11000174). The Akidawara and Satojiman alleles at the four QTLs improved the eating quality.

### QTLs detected in 91 lines in 2014

We detected 45 QTLs for eating quality in the 91 lines (Table 1, Fig. 2B). Five QTLs (*qGL1-1*, *qTA1-1*, *qST1-1*, *qHA1-1*, and *qOE1-1*) were detected on the short arm of chr. 1 (near SNP markers NIAS_Os_aa01001503 and NIAS_Os_aa01003605). Three QTLs (*qGL2-1*, *qTA2-1*, and *qOE2-1*) were detected on the short arm of chr. 2 (near NIAS_Os_aa02000675). Five QTLs (*qGL2-2*, *qTA2-2*, *qST2-2*, *qHA2-2*, and *qOE2-2*) were detected on the long arm of chr. 2 (near NIAS_Os_aa02003253). Four QTLs (*qTA8-1*, *qST8-1*, *qHA8-1*, and *qOE8-1*) were detected on the short arm of chr. 8 (near NIAS_Os_aa08001325). Five QTLs (*qGL11-1*, *qTA11-1*, *qST11-1*, *qHA11-1*, and *qOE11-1*) were detected on the short arm of chr. 11 (near NIAS_Os_ab11000174). The Akidawara and Satojiman alleles at all QTLs improved the eating quality. In addition, three QTLs (*qTA4-1*, *qST4-1*, and *qOE4-1*) were detected on the short arm of chr. 4 (near SNP markers NIAS_Os_aa04000030 and NIAS_Os_aa04000040). Five QTLs (*qGL4-3*, *qTA4-3*, *qST4-3*, *qHA4-3*, and *qOE4-3*) were detected on the distal end of the long arm of chr. 4 (near NIAS_Os_aa04009829). Five QTLs (*qGL11-3*, *qTA11-3*, *qST11-3*, *qHA11-3*, and *qOE11-3*) were detected on the long arm of chr. 11 (near NIAS_Os_aa11004506 and NIAS_Os_aa11004510). The Satojiman alleles at these QTLs improved the eating quality. Five additional QTLs, *qGL4-2*, *qTA4-2*, *qST4-2*, *qHA4-2*, and *qOE4-2*, were detected on the middle of the long arm of chr. 4 (near SNP marker NIAS_Os_aa04008763). Five QTLs, *qGL9-1*, *qTA9-1*, *qST9-1*, *qHA9-1*, and *qOE9-1*, were detected on the short arm of chr. 9 (near NIAS_Os_aa09000038). The Akidawara, Satojiman, and Ikuhikari alleles at two QTLs improved the eating quality.

One QTL for HD, *qHD1-1*, was detected on the short arm of chr. 1 (near SNP marker NIAS_Os_aa01003605) (Fig. 2B, Table 1). The Akidawara and Satojiman alleles at the QTL increased days to heading. In addition, one QTL, *qHD4-2*, was detected on the middle of the long arm of chr. 4 (near SNP marker NIAS_Os_aa04008763). The Akidawara, Satojiman, and Ikuhikari alleles at the QTL decreased days to heading. One additional QTL, *qHD6-1*, was detected on the short arm of chr. 6 (near SNP marker NIAS_Os_ac06000589). The Akidawara, Satojiman, Ikuhikari, and Koshihikari alleles at the QTL increased days to heading. Furthermore, one QTL, *qHD7-1*, was detected on the distal end of the long arm of chr. 7 (near SNP marker NIAS_Os_aa07007493). The Akidawara, Satojiman, and Ikuhikari alleles at the QTL increased days to heading. Also, one QTL, *qHD12-1*, was detected on the long arm of chr. 12 (near SNP marker NIAS_Os_aa12005256). The Satojiman alleles at the QTL increased days to heading.

### Eating quality of CSSLs

**Fig. 3.**
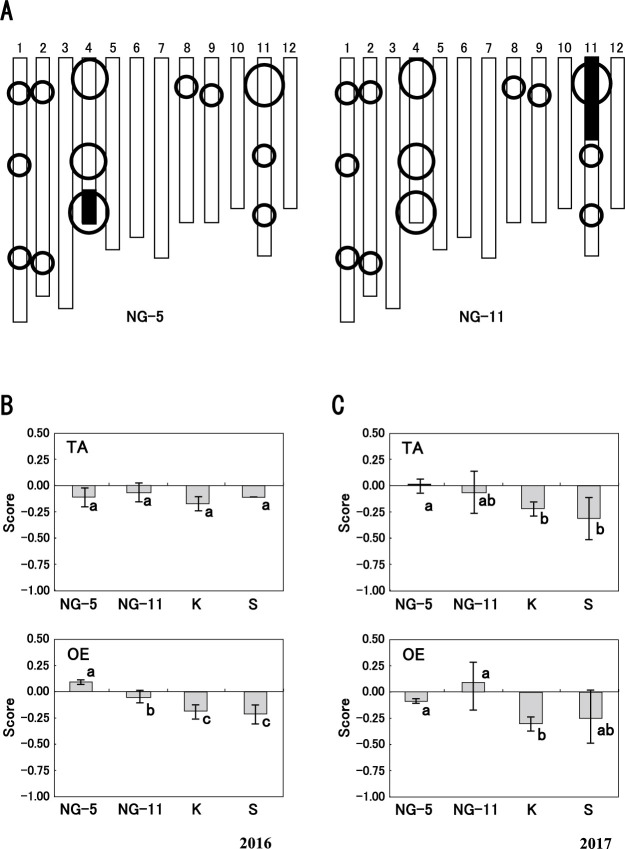
Graphical representation of genotype of two chromosome segment substitution lines, NG-5 and NG-11 (A) and scores of eating quality (B). A: Twelve *blocks* represent the chromosomes, numbered at the top. *Black* and *white* blocks denote regions derived from Satojiman and Koshihikari, respectively. *Circles* indicate eating quality QTLs identified in 68 lines and 91 lines. B: Mean scores of taste (TA) and overall evaluation (OE) of NG-5 and NG-11 in relation to Koshihikari (K) and Satojiman (S) in 2016. Eating quality of each line represents the mean score of two replications. *Error bars* indicate SD. Means followed by different letters are significantly different by *t*-test (*P* < 0.05). C: Mean scores of taste (TA) and overall evaluation (OE) of NG-5 and NG-11 in relation to Koshihikari (K) and Satojiman (S) in 2017. Eating quality of each line represents the mean score of two replications. *Error bars* indicate SD. Means followed by different letters are significantly different by *t*-test (*P* < 0.10).

To confirm the presence of the QTL on the distal end of the long arm of chr. 4 and that on the short arm of chr. 11, we selected each CSSL from advanced backcross progeny (Fig. 3A). Analysis of 1297 SNP markers confirmed that a relatively short chromosome segment of Satojiman carrying one QTL for each CSSL was substituted in the genetic background of Koshihikari. All other SNP markers were homozygous for Koshihikari, indicating that the insertion of any other Satojiman segments was unlikely.

In both years, the GL scores of a NG-5 (0.03 in 2016 and –0.07 in 2017) were almost the same as those of Koshihikari (0.07 and –0.21) (Supplemental Fig. 1). The ST, TA, and HA scores of a NG-5 (0.13 for ST, –0.11 for TA, and 0.15 for HA) were slightly higher than those of Koshihikari (0.07 for ST, –0.16 for TA, and 0.06 for HA, respectively) in 2016 (Fig. 3B, Supplemental Fig. 1A). In 2017, ST score of a NG-5 (–0.14) was lower than that of Koshihikari (0.02) (*P* < 0.05). In 2017, the TA score of a NG-5 (0.00) was higher than that of Koshihikari (–0.20) (*P* < 0.05) (Fig. 3C). The HA score of a NG-5 (0.07) was almost the same as that of Koshihikari (0.27) in 2017. The OE scores of a NG-5 (0.05 in 2016 and –0.07 in 2017) were higher than those of Koshihikari (–0.18 and –0.31) (*P* < 0.05) (Fig. 3).

For a NG-11, the GL score (0.04) was almost the same as that of Koshihikari (0.07) in 2016 (Supplemental Fig. 1A). In 2017, the GL score (0.10) of a NG-11 was higher than that of Koshihikari (–0.21) (*P* < 0.05) (Supplemental Fig. 1B). The ST scores (0.02 in 2016 and 0.31 in 2017) of a NG-11 were almost the same as those of Koshihikari (0.07 and 0.02) (Supplemental Fig. 1). The TA score (–0.02 in 2016 and –0.08 in 2017) were slightly higher than those of Koshihikari (–0.16 and –0.20) (Fig. 3). The HA scores (–0.04 in 2016 and 0.12 in 2017) were almost the same as those of Koshihikari (0.15 and 0.27) (Supplemental Fig. 1). The OE scores of a NG-11 (–0.03 in 2016 and 0.08 in 2017) were higher than those of Koshihikari (–0.18 and –0.31) (*P* < 0.10) (Fig. 3). These results clearly verify the effects of the Satojiman allele at these QTLs on the end of the long arm of chr. 4 and on the short arm of chr. 11.

The AC and PC values of NG-5 (17.4% for AC and 5.3% for PC) and NG-11 (17.0% for AC and 5.4% for PC) were almost the same as those of Koshihikari (17.2% for AC and 5.0% for PC) (Supplemental Fig. 1).

## Discussion

Eating quality is an important trait in rice breeding in Japan. As consumers prefer cultivars such as Koshihikari, Akidawara, Satojiman, and Ikuhikari, selection for eating quality has focused on characteristics of these cultivars, so, these cultivars have been extensively used as parental lines in most breeding programs in Japan.

The eating quality of breeding materials is usually evaluated by sensory test. Since evaluation should be done on advanced generations, such as F_6_ or later, it is very time consuming and labor intensive. Other evaluation and selection methods, such as measuring chemical components and MAS, would be required to improve selection. In this regard, in order to establish MAS for eating quality, we have been interested in identifying chromosomal regions affecting eating quality.

Takeuchi *et al.* (2008) performed QTL analysis using two BILs derived from crosses of Nipponbare (which has inferior eating quality) /Koshihikari//Nipponbare and Nipponbare/Koshihikari//Koshihikari. And detected one major QTL for eating quality on the distal end of the short arm of chr. 3, at which the Koshihikari alleles increased eating quality. In this study, we confirmed by using DNA markers that Akidawara, Satojiman, and Ikuhikari have the Koshihikari alleles at the eating quality QTL of the distal end of the short arm of chr. 3 (data not shown). On the other hand, we thought that its QTLs do not explain all of the differences in eating quality among Akidawara, Satojiman, and Ikuhikari. Thus, genetic dissection of eating quality among these cultivars remains to be clarified.

In this study, we identified four common chromosomal regions involved in eating quality on the short arm of chr. 4, the middle of the long arm of chr. 4, the distal end of the long arm chr. 4, and the short arm of chr. 11 by genome-wide association (GWAS) mapping using two breeding populations. Interestingly, several QTLs for the components of eating quality are clustered in these regions. Consistent detection of these QTLs in different mapping populations in two consecutive years suggests that they are stably expressed in different breeding population and under different environmental conditions. A QTL region on short arm of chr. 4 had been reported previously. Another QTL on the middle of the long arm of chr. 4 might be affected by heading date. The other QTLs at the distal end of the long arm of chr. 4 and the short arm of chr. 11 were not identified previously. Furthermore, their genetic effects were verified by the development of each CSSL for the distal end of the long arm of chr. 4 and the short arm of chr. 11.

The mapping resolution of the two breeding populations used in this study made it difficult to conclude whether these apparent QTLs represent pleiotropy of just one QTL, or are tightly linked but different QTLs. Other studies have also identified QTLs for multiple components of eating quality (Takeuchi *et al.* 2008, Tanaka *et al.* 2006, Wan *et al.* 2004). Although the individual components of eating quality (GL, TA, ST, and HA) seem to be different characteristics, they are related to each other (Takeuchi *et al.* 2008). High-resolution substitution mapping should reveal this.

Previous study reported several QTLs for eating quality on the short arm of chr. 4. Wada *et al.* (2008) identified two QTL for appearance, *qOE4* and *qST4*, in the center of the short arm of chr. 4 in a recombinant inbred lines derived from a cross between two *japonica* cultivars ‘Moritawase’ and Koshihikari. The three QTLs on the short arm of chr. 4 we detected here (*qOE4-1*, *qTA4-1*, and *qST4-1*) appear to coincide with the two QTLs, *qOE4* and *qST4*, identified by Wada *et al.* (2008). Previous studies reported several QTLs for eating quality on chr. 11. Takeuchi *et al.* (2008) identified one QTL for appearance, *qTA11*, in the centromere region of chr. 11 in BILs derived from crosses of two *japonica* cultivars (Nipponbare/Koshihikari//Nipponbare). Wada *et al.* (2008) also identified one QTL for eating quality, *qGL11*, in centromere region of chr. 11. The five QTLs on the short arm of chr. 11 we detected here (*qOE11-1*, *qGL11-1*, *qTA11-1*, *qST11-1*, and *qHA11-1*) appear to be different loci from the two QTLs, *qTA11* and *qGL11*, identified by Takeuchi *et al.* (2008) and Wada *et al.* (2008), respectively. Further analysis, including fine mapping and cloning of genes at these QTLs, should be conducted to clarify the relationships between the QTLs detected in this study and other eating quality QTLs.

QTLs on the distal end of the long arm of chr. 4 and the short arm of chr. 11 were commonly identified in 2013 and 2014 in breeding populations, and in 2016 and 2017 in the CSSLs. This result suggests that the effects of these QTLs are reproducible in different genetic backgrounds of Koshihikari and other breeding lines. The average temperature of ripening period was abnormally high in August in 2013, 2016, and 2017. The high temperature would affect the eating quality of Koshihikari. This result suggests that the effects of these QTLs are reproducible in different environmental conditions too.

It is well known that chemical properties, including AC and PC, affect eating quality (Ishima *et al.* 1974, Juliano *et al.* 1965). In this study, the AC and PC values of NG-5 (with QTLs on the distal end of the long arm of chr. 4) and NG-11 (with QTLs on the short arm of chr. 11) were almost the same as those of Koshihikari. These results suggest that the higher eating quality due to the Satojiman alleles at QTLs on the distal end of the long arm of chr. 4 and the short arm of chr. 11 was not related to the AC and PC in endosperm.

In general, eating quality was affected by heading date. Components of eating quality such as GL and ST were positively correlated with heading date (Tanaka *et al.* 2006). In this study, we identified five QTLs for heading date (*qHD1-1*, *qHD4-2*, *qHD6-1*, *qHD7-1*, and *qHD12-1*) on the short arm of chr. 1, the middle of the long arm of chr. 4, the short arm of chr. 6, the distal end of the long arm of chr. 7, and the long arm of chr. 12 by GWAS using 91 lines in 2014. The five QTLs, we detected here (*qHD1-1*, *qHD4-2*, *qHD6-1*, *qHD7-1*, and *qHD12-1*), appear to coincide with five QTL (*qDTH1-1*, *qDTH4.5*, *Hd-1*, *Hd-2*, and *Hd13*), respectively identified by Kitazawa *et al.* (2024) and Ogiso-Tanaka *et al.* (2017). In this study, two chromosomal regions for eating quality, on the short arm of chr. 1 and the middle of the long arm of chr. 4, were the same as those of two QTLs for heading date, *qHD1-1* and *qHD4-2*. These results indicate that QTLs for eating quality on the short arm of chr. 1 and on the middle of the long arm of chr. 4 were detected by the effect of the variation in heading date.

In rice breeding, sensory tests of advanced lines are time consuming and labor intensive. It is also difficult to establish standards for eating quality owing to the variable reliability of sensory tests, which depend on the talents of panelists. Establishing more effective and reliable methods of screening for eating quality will become a very important task in future rice breeding in Japan. MAS is one solution, using DNA markers NIAS_Os_aa04009569, NIAS_Os_aa04009710, NIAS_Os_aa04009737, and NIAS_Os_aa04009829 (on the distal end of the long arm of chr. 4) and NIAS_Os_ab11000174 (on the short arm of chr. 11), located near the QTLs for eating quality. To evaluate the potential of these markers for indirect selection, researchers should investigate the allelic frequency of these markers among elite cultivars with good eating quality developed recently in Japan.

In the last score, several QTLs with relatively large phenotypic effects have been cloned by map-based strategies (Yamamoto and Yano 2008, Yano 2001). Molecular identification of such genes has brought new insights into phenotypic traits, such as stress tolerance and yield potential (Ashikari *et al.* 2005, Ren *et al.* 2005). As long as eating quality of cooked rice is evaluated by sensory testing, it will be difficult to reveal the genetic basis of components of eating quality, except in terms of chemicals such as amylose and protein. Here, we identified QTLs with relatively good phenotypic effects on eating quality (the alleles from Satojiman increased the level of eating quality of Koshihikari) independent of AC. This will provide an opportunity to clone the genes involved in eating quality. Molecular identification of such genes will help to create new methods of evaluation and selection in rice breeding.

## Author Contribution Statement

Y.T, T.Y., J.Y., and I.A. designed the experiments. Y.T., Y.K., M.K., A.G., K.M., H.S., H.H., N.K., M.Y., T.I., and I.A. generated the breeding materials and evaluated eating quality of cooked rice. Y.T., T.Y., J.Y., and S.F. carried out the GWAS analysis. Y.T. wrote the manuscript.

## Supplementary Material

Supplemental Figure

Supplemental Table

## Figures and Tables

**Table 1.** Putative QTLs controlling the eating quality detected in GWAS

Year	QTL*^a^*	Chr.	Nearest SNP marker	Peak SNP marker position (bp)*^b^*	LOD	*P*-value	Allele*^c^*	Mean of severity score			
								Akidawara allele	Satojiman allele	Ikuhikari allele	Koshihikari allele
*2013*	*qGL1-2*	1	NIAS_Os_aa01005142	4838836	4.27	0.00005	S	–0.63	–0.23	–0.63	–0.63
	*qTA1-2*	1	NIAS_Os_aa01005142	4838836	5.44	0.00001	S	–0.80	–0.44	–0.80	–0.80
	*qST1-2*	1	NIAS_Os_aa01005142	4838836	6.58	0.00001	S	–0.71	–0.19	–0.71	–0.71
	*qHA1-2*	1	NIAS_Os_aa01005142	4838836	3.24	0.00058	S	0.44	0.13	0.44	0.44
	*qOE1-2*	1	NIAS_Os_aa01005142	4838836	5.14	0.00001	S	–0.99	–0.49	–0.99	–0.99
*2013*	*qGL1-3*	1	NIAS_Os_aa01009984	28607327	4.23	0.00006	AI	–0.20	–0.59	–0.20	–0.59
	*qTA1-3*	1	NIAS_Os_aa01009984	28607327	4.75	0.00002	AI	–0.42	–0.76	–0.42	–0.76
	*qST1-3*	1	NIAS_Os_aa01009984	28607327	4.51	0.00003	AI	–0.21	–0.64	–0.21	–0.64
	*qOE1-3*	1	NIAS_Os_aa01009984	28607327	4.29	0.00005	AI	–0.48	–0.93	–0.48	–0.93
*2013*	*qGL4-1*	4	NIAS_Os_aa04000030	773152	4.58	0.00003	S	–0.65	–0.23	–0.65	–0.65
	*qTA4-1*	4	NIAS_Os_aa04000030	773152	4.99	0.00001	S	–0.80	–0.45	–0.80	–0.80
	*qST4-1*	4	NIAS_Os_aa04000030	773152	5.61	0.00001	S	–0.70	–0.22	–0.70	–0.70
	*qOE4-1*	4	NIAS_Os_aa04000030	773152	4.70	0.00002	S	–1.00	–0.50	–1.00	–1.00
*2013*	*qST4-2*	4	NIAS_Os_aa04008763	31406658	3.13	0.00075	ASI	–0.30	–0.30	–0.30	–0.70
	*qHA4-2*	4	NIAS_Os_aa04008763	31406658	4.13	0.00007	ASI	0.16	0.16	0.16	0.50
*2013*	*qGL4-3*	4	NIAS_Os_aa04009710	34146238	4.62	0.00002	S	–0.71	–0.26	–0.71	–0.71
	*qTA4-3*	4	NIAS_Os_aa04009737	34529440	4.00	0.00010	S	–0.78	–0.49	–0.78	–0.78
	*qST4-3*	4	NIAS_Os_aa04009569	33225262	4.64	0.00002	S	–0.72	–0.24	–0.72	–0.72
	*qOE4-3*	4	NIAS_Os_aa04009737	34529440	3.42	0.00038	S	–0.97	–0.56	–0.97	–0.97
*2013*	*qGL11-1*	11	NIAS_Os_ab11000174	5018628	3.59	0.00026	AS	–0.26	–0.26	–0.62	–0.62
	*qTA11-1*	11	NIAS_Os_ab11000174	5018628	4.76	0.00002	AS	–0.49	–0.49	–0.75	–0.75
	*qST11-1*	11	NIAS_Os_ab11000174	5018628	4.58	0.00003	AS	–0.26	–0.26	–0.66	–0.66
	*qOE11-1*	11	NIAS_Os_ab11000174	5018628	4.28	0.00005	AS	–0.55	–0.55	–0.94	–0.94
*2013*	*qGL11-2*	11	NIAS_Os_aa11003316	15976536	4.09	0.00008	S	–0.67	–0.24	–0.67	–0.67
	*qTA11-2*	11	NIAS_Os_aa11003316	15976536	5.06	0.00001	S	–0.79	–0.47	–0.79	–0.79
	*qST11-2*	11	NIAS_Os_aa11003316	15976536	4.28	0.00005	S	–0.70	–0.25	–0.70	–0.70
	*qOE11-2*	11	NIAS_Os_aa11003316	15976536	4.40	0.00004	S	–0.99	–0.52	–0.99	–0.99
*2014*	*qGL1-1*	1	NIAS_Os_aa01003605	3028485	3.15	0.00071	AS	–0.14	–0.14	–0.4	–0.4
	*qTA1-1*	1	NIAS_Os_aa01003605	3028485	3.48	0.00033	AS	–0.35	–0.35	–0.55	–0.55
	*qST1-1*	1	NIAS_Os_aa01003605	3028485	3.68	0.00021	AS	–0.20	–0.20	–0.47	–0.47
	*qHA1-1*	1	NIAS_Os_aa01003605	3028485	4.14	0.00007	AS	0.01	0.01	0.29	0.29
	*qOE1-1*	1	NIAS_Os_aa01001503	2386351	3.20	0.00063	AS	–0.41	–0.41	–0.71	–0.71
	*qHD1-1*	1	NIAS_Os_aa01003605	3028485	3.06	0.00087	AS	113.0	113.0	109.1	109.1
*2014*	*qGL2-1*	2	NIAS_Os_aa02000675	5100629	3.31	0.00048	AS	–0.37	–0.37	–0.67	–0.67
	*qTA2-1*	2	NIAS_Os_aa02000675	5100629	4.33	0.00005	AS	–0.37	–0.37	–0.67	–0.67
	*qOE2-1*	2	NIAS_Os_aa02000675	5100629	3.56	0.00027	AS	–0.37	–0.37	–0.67	–0.67
*2014*	*qGL2-2*	2	NIAS_Os_aa02003253	28181279	4.94	0.00001	AS	–0.14	–0.14	–0.45	–0.45
	*qTA2-2*	2	NIAS_Os_aa02003253	28181279	6.07	0.00001	AS	–0.31	–0.31	–0.66	–0.66
	*qST2-2*	2	NIAS_Os_aa02003253	28181279	5.09	0.00001	AS	–0.17	–0.17	–0.56	–0.56
	*qHA2-2*	2	NIAS_Os_aa02003253	28181279	3.50	0.00032	AS	0.03	0.03	0.29	0.29
	*qOE2-2*	2	NIAS_Os_aa02003253	28181279	6.90	0.00001	AS	–0.36	–0.36	–0.85	–0.85
*2014*	*qTA4-1*	4	NIAS_Os_aa04000030	773152	4.66	0.00002	S	–0.53	–0.31	–0.53	–0.53
	*qST4-1*	4	NIAS_Os_aa04000030	773152	3.19	0.00064	S	–0.43	–0.17	–0.43	–0.43
	*qOE4-1*	4	NIAS_Os_aa04000040	1075655	4.44	0.00004	S	–0.68	–0.36	–0.68	–0.68
*2014*	*qGL4-2*	4	NIAS_Os_aa04008763	31406658	5.63	0.00001	ASI	–0.14	–0.14	–0.14	–0.53
	*qTA4-2*	4	NIAS_Os_aa04008763	31406658	6.47	0.00001	ASI	–0.31	–0.31	–0.31	–0.74
	*qST4-2*	4	NIAS_Os_aa04008763	31406658	5.44	0.00001	ASI	–0.19	–0.19	–0.19	–0.63
	*qHA4-2*	4	NIAS_Os_aa04008763	31406658	4.17	0.00007	ASI	0.02	0.02	0.02	0.38
	*qOE4-2*	4	NIAS_Os_aa04008763	31406658	6.45	0.00001	ASI	–0.37	–0.37	–0.37	–0.95
	*qHD4-2*	4	NIAS_Os_aa04008763	31406658	3.28	0.00053	ASI	109.9	109.9	109.9	114.3
*2014*	*qGL4-3*	4	NIAS_Os_aa04009829	34992381	5.35	0.00001	S	–0.52	–0.11	–0.52	–0.52
	*qTA4-3*	4	NIAS_Os_aa04009829	34992381	6.54	0.00001	S	–0.72	–0.29	–0.72	–0.72
	*qST4-3*	4	NIAS_Os_aa04009829	34992381	6.36	0.00001	S	–0.63	–0.15	–0.63	–0.63
	*qHA4-3*	4	NIAS_Os_aa04009829	34992381	3.95	0.00011	S	0.37	0.00	0.37	0.37
	*qOE4-3*	4	NIAS_Os_aa04009829	34992381	6.81	0.00001	S	–0.94	–0.33	–0.94	–0.94
*2014*	*qHD6-1*	6	NIAS_Os_ac06000589	11145980	6.20	0.00001	ASIK	112.6	112.6	112.6	112.6
*2014*	*qHD7-1*	7	NIAS_Os_aa07007493	27485237	4.80	0.00002	ASI	112.9	112.9	112.9	101.5
*2014*	*qTA8-1*	8	NIAS_Os_aa08001325	7384130	4.90	0.00001	AS	–0.34	–0.34	–0.60	–0.60
	*qST8-1*	8	NIAS_Os_aa08001325	7384130	4.94	0.00001	AS	–0.20	–0.20	–0.52	–0.52
	*qHA8-1*	8	NIAS_Os_aa08001325	7384130	5.12	0.00001	AS	0.01	0.01	0.33	0.33
	*qOE8-1*	8	NIAS_Os_aa08001325	7384130	5.10	0.00001	AS	–0.40	–0.40	–0.76	–0.76
*2014*	*qGL9-1*	9	NIAS_Os_aa09000038	9071039	3.70	0.00020	ASI	–0.15	–0.15	–0.15	–0.53
	*qTA9-1*	9	NIAS_Os_aa09000038	9071039	4.20	0.00006	ASI	–0.32	–0.32	–0.32	–0.73
	*qST9-1*	9	NIAS_Os_aa09000038	9071039	3.39	0.00041	ASI	–0.19	–0.19	–0.19	–0.62
	*qHA9-1*	9	NIAS_Os_aa09000038	9071039	3.53	0.00029	ASI	0.03	0.03	0.03	0.39
	*qOE9-1*	9	NIAS_Os_aa09000038	9071039	4.90	0.00001	ASI	–0.38	–0.38	–0.38	–0.96
*2014*	*qGL11-1*	11	NIAS_Os_ab11000174	5018628	6.65	0.00001	AS	–0.11	–0.11	–0.52	–0.52
	*qTA11-1*	11	NIAS_Os_ab11000174	5018628	7.36	0.00001	AS	–0.29	–0.29	–0.69	–0.69
	*qST11-1*	11	NIAS_Os_ab11000174	5018628	7.27	0.00001	AS	–0.16	–0.16	–0.61	–0.61
	*qHA11-1*	11	NIAS_Os_ab11000174	5018628	5.56	0.00001	AS	–0.03	–0.03	0.40	0.40
	*qOE11-1*	11	NIAS_Os_ab11000174	5018628	7.00	0.00001	AS	–0.34	–0.34	–0.89	–0.89
*2014*	*qGL11-3*	11	NIAS_Os_aa11004510	23738909	3.49	0.00033	S	–0.38	–0.16	–0.38	–0.38
	*qTA11-3*	11	NIAS_Os_aa11004510	23738909	7.36	0.00001	S	–0.60	–0.31	–0.60	–0.60
	*qST11-3*	11	NIAS_Os_aa11004506	21888015	5.62	0.00001	S	–0.53	–0.20	–0.53	–0.53
	*qHA11-3*	11	NIAS_Os_aa11004510	23738909	3.56	0.00028	S	–0.75	–0.37	–0.75	–0.75
	*qOE11-3*	11	NIAS_Os_aa11004510	23738909	6.41	0.00001	S	–0.75	–0.37	–0.75	–0.75
*2014*	*qHD12-1*	12	NIAS_Os_aa12005256	25506887	7.42	0.00001	S	106.6	113.8	106.6	106.6

*^a^* OE, overall evaluation; GL, glossiness; TA, taste; ST, stickiness; HA, hardness; HD, days to heading. *^b^* The position of each SNP marker was based on the genome sequence of Nipponbare (build 1.0). *^c^* The A (Akidawara), S (Satojiman), and/or I (Ikuhikari) allele(s) at QTL increased eating quality. Or, the A (Akidawara), S (Satojiman), I (Ikuhikari), and/or K (Koshihikari) allele(s) at QTL increased days to heading.

## References

[B1] Ando, I., H. Nemoto, H. Kato, H. Ohta, H. Hirabayashi, Y. Takeuchi, H. Sato, T. Ishii, H. Maeda, T. Imbe et al. (2011) A new rice cultivar “Akidawara” with high yield, good grain appearance, and good eating quality. Breed Res 13: 35–41 (in Japanese with English summary).

[B2] Ashikari, M., H. Sakakibara, S. Lin, T. Yamamoto, T. Takashi, A. Nishimura, E.R. Angeles, Q. Qian, H. Kitano and M. Matsuoka (2005) Cytokinin oxidase regulates rice grain production. Science 29: 741–745. 10.1126/science.1113373 15976269

[B3] Bett-Garber, K.L., E.T. Champagne, A.M. McClung, K.A. Moldenhauer, S.D. Linscombe and K.S. McKenzie (2001) Categorizing rice cultivars based on cluster analysis of amylose content, protein content and sensory attributes. Cereal Chem 78: 551–558.

[B4] Hayashi, K., N. Hashimoto, M. Daigen and I. Ashikawa (2004) Development of PCR-based SNP markers for rice blast resistance genes at the *Piz* locus. Theor Appl Genet 108: 1212–1220. 14740086 10.1007/s00122-003-1553-0

[B5] Ishima, T., H. Taira, H. Taira and K. Mikoshiba (1974) Effect of nitrogenous fertilizer application and protein content in milled rice on organoleptic quality of cooked rice. Report of National Food Research Institute 29: 9–15 (in Japanese with English summary).

[B6] Juliano, B.O. (1971) A simplified assay for milled-rice amylose. Cereal Science Today 16: 334–340.

[B7] Juliano, B.O. (1985) Criteria and test for rice grain quality. *In*: Juliano, B.O. (ed.) Rice: chemistry and technology. American Association of Cereal Chemists, St. Paul, MN, USA, pp. 443–513.

[B8] Juliano, B.O., L.U. Oñate and A.M. del Mundo (1965) Relation of starch composition, protein content, and gelatinization temperature to cooking and eating qualities of milled rice. Food Technol 19: 1006–1011.

[B9] Kitazawa, N., A. Shomura, T. Mizubayashi, T. Ando, N. Hayashi, S. Yabe, K. Matsubara, K. Ebana, U. Yamanouchi and S. Fukuoka. (2024) Development of SNP genotyping assays for heading date in rice. Breed Sci 74: 274–284. 39555007 10.1270/jsbbs.23093 PMC11561416

[B10] Kruglyak, L. and E.S. Lander (1995) A nonparametric approach for mapping quantitative trait loci. Genetics 139: 1421–1428. 7768449 10.1093/genetics/139.3.1421 PMC1206467

[B11] Nagasaki, H., K. Ebana, T. Shibaya, J. Yonemaru and M. Yano (2010) Core single-nucleotide polymorphisms—a tool for genetic analysis of the Japanese rice population. Breed Sci 60: 648–655.

[B12] Neeraja, C.N., R. Maghirang-Rodriguez, A. Pamplona, S. Heuer, B.C.Y. Collard, E.M. Septiningsih, G. Vergara, D. Sanchez, K. Xu, A.M. Ismail et al. (2007) A marker-assisted backcross approach for developing submergence-tolerant rice cultivars. Theor Appl Genet 115: 767–776. 17657470 10.1007/s00122-007-0607-0

[B13] Ogiso-Tanaka, E., T. Tanaka, K. Tanaka, Y. Nonoue, T. Sasaki, E. Fushimi, Y. Koide, Y. Okumoto, M. Yano and H. Saito (2017) Detection of novel QTLs *qDTH4.5* and *qDTH6.3*, which confer late heading under short-day conditions, by SSR marker-based and QTL-seq analysis. Breed Sci 67: 101–109. 28588386 10.1270/jsbbs.16096 PMC5445965

[B14] Ren, Z.H., J.P. Gao, L.G. Li, X.L. Cai, W. Huang, D.Y. Chao, M.Z. Zhu, Z.Y. Wang, S. Luan and H.X. Lin (2005) A rice quantitative trait locus for salt tolerance encodes a sodium transporter. Nat Genet 37: 1141–1146. 16155566 10.1038/ng1643

[B15] Sato, H., Y. Suzuki, M. Sakai and T. Imbe (2002) Molecular characterization of *Wx-mq*, a novel mutant gene for low-amylose content in endosperm of rice (*Oryza sativa* L.). Breed Sci 52: 131–135.

[B16] Sato, H., T. Imbe, I. Ando, N. Horisue, H. Nemoto, M. Sakai, H. Ohta, H. Hirabayashi, O. Ideta, M. Takadate et al. (2013) Breeding of the rice variety “Satojiman” with high eating quality and resistance to the rice stripe virus. Bulletin of the National Institute of Crop Science 14: 37–56.

[B17] Singh, S., J.S. Sidhu, N. Huang, Y. Vikal, Z. Li, D.S. Brar, H.S. Dhaliwal and G.S. Khush (2001) Pyramiding three bacterial blight resistance genes (*xa5*, *xa13* and *Xa21*) using marker-assisted selection into indica rice cultivar PR106. Theor Appl Genet 102: 1011–1015.

[B18] Sugiura, N., T. Tsuji, K. Fujii, T. Kato, N. Saka, T. Touyama, Y. Hayano-Saito and T. Izawa (2004) Molecular marker-assisted selection in a recurrent backcross breeding for the incorporation of resistance to rice stripe virus and panicle blast in rice (*Oryza* *sativa* L.). Breed Res 6: 143–148 (in Japanese).

[B19] Suzuki, Y., H.Y. Hirano, Y. Sano, U. Matsukura, S. Kawasaki, M. Chono, S. Nakamura and H. Sato (2003) Isolation and characterization of a rice mutant with enhanced amylose content in endosperm derived from a low amylose variety ‘Snow pearl’. Rice Genet Newsl 20: 65–66.

[B20] Takeuchi, Y., T. Ebitani, T. Yamamoto, H. Sato, H. Ohta, H. Hirabayashi, H. Kato, I. Ando, H. Nemoto, T. Imbe et al. (2006) Development of isogenic lines of rice cultivar Koshihikari with early and late heading by marker-assisted selection. Breed Sci 56: 405–413.

[B21] Takeuchi, Y., Y. Nonoue, T. Ebitani, K. Suzuki, N. Aoki, H. Sato, O. Ideta, H. Hirabayashi, M. Hirayama, H. Ohta et al. (2007) QTL detection for eating quality including glossiness, stickiness, taste and hardness of cooked rice. Breed Sci 57: 231–242.

[B22] Takeuchi, Y., K. Hori, K. Suzuki, Y. Nonoue, Y. Takemoto-Kuno, H. Maeda, H. Sato, H. Hirabayashi, H. Ohta, T. Ishii et al. (2008) Major QTLs for eating quality of an elite Japanese rice cultivar, Koshihikari, on the short arm of chromosome 3. Breed Sci 58: 437–445.

[B23] Tanaka, I., A. Kobayashi, K. Tomita, Y. Takeuchi, M. Yamagishi, M. Yano, T. Sasaki and H. Horiuchi (2006) Detection of quantitative trait loci for stickiness and appearance based on eating quality test in *japonica* rice cultivar. Breed Res 8: 39–47 (in Japanese with English summary).

[B24] Tomita, K., H. Horiuchi, K. Terada, M. Tanoi, A. Kobayashi, I. Tanaka, T. Minobe, H. Furuta, A. Yamamoto, H. Shinoyama et al. (2005) “Ikuhikari”, a new rice cultivar. Bulletin of Fukui Agricultural Experiment Station 42: 1–15 (in Japanese with English summary).

[B25] Wada, T., T. Ogata, M. Tsubone, Y. Uchimura and Y. Matsue (2008) Mapping of QTLs for eating quality and physicochemical properties of the *japonica* rice ‘Koshihikari’. Breed Sci 58: 427–435.

[B26] Wan, X.Y., J.M. Wan, C.C. Su, C.M. Wang, W.B. Shen, J.M. Li, H.L. Wang, L. Jiang, S.J. Liu, L.M. Chen et al. (2004) QTL detection for eating quality of cooked rice in a population of chromosome segment substitution lines. Theor Appl Genet 110: 71–79. 15551043 10.1007/s00122-004-1744-3

[B27] Wang, Z.X., S. Sakaguchi, Y. Oka, N. Kitazawa and Y. Minobe (2005) Breeding of semi-dwarf Koshihikari by using genomic breeding method. Breed Res 7 (Suppl. 1 and 2): 217 (in Japanese).

[B28] Yamamoto, T., N. Horisue and R. Ikeda (1996) Rice Breeding Manual. Yokendo Ltd., Tokyo, pp. 74–76 (in Japanese).

[B29] Yamamoto, T. and M. Yano (2008) Detection and molecular cloning of genes underlying quantitative phenotypic variations in rice. *In*: Hirano, H.Y., A. Hirai, Y. Sano and T. Sasaki (eds.) Rice Biology in the Genomics Era, Springer, Heidelberg, pp. 295–308.

[B30] Yamamoto, T., J. Yonemaru and M. Yano (2009) Towards the understanding of complex traits in rice: substantially or superficially? DNA Res 16: 141–154. 19359285 10.1093/dnares/dsp006 PMC2695773

[B31] Yamamoto, T., H. Nagasaki, J. Yonemaru, K. Ebana, M. Nakajima, T. Shibaya and M. Yano (2010) Fine definition of the pedigree haplotypes of closely related rice cultivars by means of genome-wide discovery of single-nucleotide polymorphisms. BMC Genomics 11: 267. 20423466 10.1186/1471-2164-11-267 PMC2874813

[B32] Yamamoto, Y. and T. Ogawa (1992) Eating quality in Japanese rice cultivars. Japan J Breed 42: 177–183 (in Japanese).

[B33] Yano, M. (2001) Genetic and molecular dissection of naturally occurring variation. Curr Opin Plant Biol 4: 130–135. 11228435 10.1016/s1369-5266(00)00148-5

