# Supplementary material for: Good-eating-quality QTLs detected in two breeding populations by genome-wide association mapping increase eating quality of the Japanese rice cultivar ‘Koshihikari’
Source: Breed Sci. 2025 Oct 24;75(5):358–68. doi: 10.1270/jsbbs.25025 (PMC13129571; doi:10.1270/jsbbs.25025)
Supplement: Supplementary file 1 — Supplemental Figure [file 75_358_s1.pdf]

doi: 10.1270/jsbbs.25025

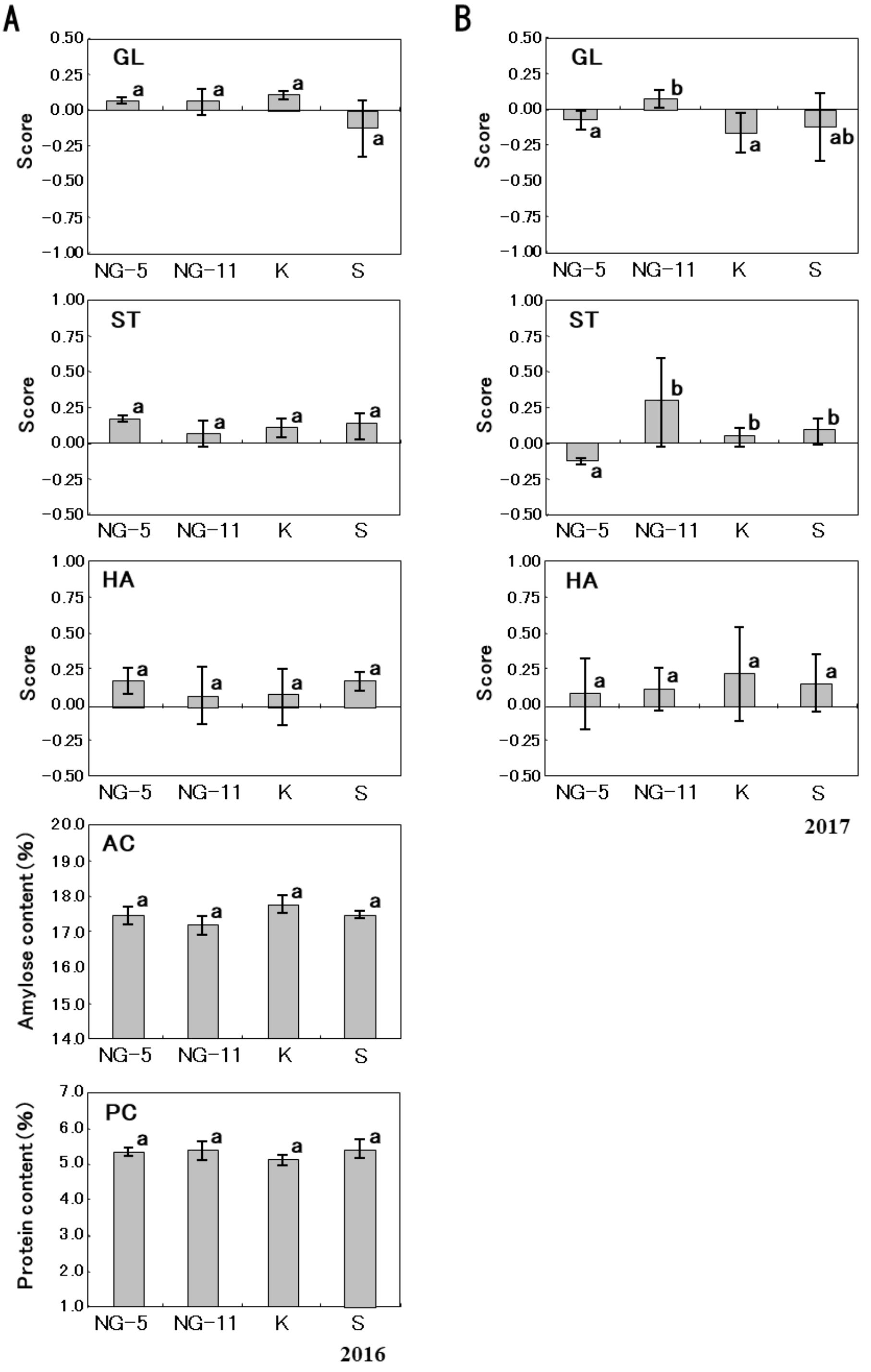

Supplemental Fig. 1 Scores of eating quality of two chromosome segment substitution lines, NG-5 and NG-11.

A: Mean scores of glossiness (GL), stickiness (ST), hardness (HA), amylose content (AC), and protein content (PC) of NG-5 and NG-11 in relation to Koshihikari (K) and

Satojiman (S) in 2016. Eating quality of each line represents the mean score of two replications. *Error bars* indicate SD. Means followed by different letters are significantly different by *t*-test ($P < 0.05$).

B: Mean scores of glossiness (GL), stickiness (ST), and hardness (HA) of NG-5 and NG-11 in relation to Koshihikari (K) and Satojiman (S) in 2017. Eating quality of each line represents the mean score of two replications. *Error bars* indicate SD. Means followed by different letters are significantly different by *t*-test ($P < 0.05$).
