# Supplementary material for: Good-eating-quality QTLs detected in two breeding populations by genome-wide association mapping increase eating quality of the Japanese rice cultivar ‘Koshihikari’
Source: Breed Sci. 2025 Oct 24;75(5):358–68. doi: 10.1270/jsbbs.25025 (PMC13129571; doi:10.1270/jsbbs.25025)
Supplement: Supplementary file 2 — Supplemental Table [file 75_358_s2.pdf]

doi: 10.1270/jsbbs.25025

Supplemental Table 1. Breeding lines used for genome-wide association QTL mapping for eating quality

Year	Serial number	Line	Cross combination [a]
2013	1	A2456	Satojiman//Ikuhikari/A1289
	2	A2454	Satojiman//Ikuhikari/A1289
	3	A2459	Satojiman//Ikuhikari/A1289
	4	A2451	Satojiman//Ikuhikari/A1289
	5	KC256	Satojiman/T57
	6	A2456	Satojiman//Ikuhikari/A1289
	7	A2417	Satojiman/T65
	8	A2418	Satojiman/T65
	9	A2419	Satojiman/T65
	10	A2454	Satojiman//Ikuhikari/A1289
	11	A2545	Satojiman//Kinuhikari/AC1072
	12	KC244	Satojiman/E171
	13	A2422	Satojiman/T65
	14	A2421	Satojiman/T65
	15	A2459	Satojiman//Ikuhikari/A1289
	16	A2451	Satojiman//Ikuhikari/A1289
	17	A2276	Satojiman//Kinuhikari/AC1072
	18	KC251	Y70/Satojiman
	19	KC252	Satojiman/I71
	20	KC238	KC199/Satojiman
	21	A2680	T71/Satojiman
	22	A2760	Satojiman/C129
	23	A2713	Satojiman/A1625
	24	A2624	Satojiman/A1628
	25	A2714	Satojiman/A1625
	26	A2751	Satojiman/Tsuyahime
	27	A2715	Satojiman/HC221
	28	A2757	Satojiman/C129
	29	A2758	Satojiman/C129
	30	A2601	KC247/Satojiman
	31	A2602	KC247/Satojiman
	32	A2681	T71/Satojiman
	33	A2752	Satojiman/C129
	34	A2753	Satojiman/C129
	35	A2754	Satojiman/C129
	36	A2755	Satojiman/C129
	37	A2756	Satojiman/C129
	38	A2603	KC247/Satojiman
	39	A2604	KC247/Satojiman
	40	A2605	KC247/Satojiman
	41	A2606	KC247/Satojiman
	42	A2607	KC247/Satojiman
	43	A2625	Satojiman/A1628
	44	A2626	Satojiman/A1628
	45	A2698	Satojiman/T65
	46	A2699	Satojiman/T65
	47	A2700	Satojiman/T65
	48	A2759	Satojiman/C129
	49	A2337	Ikuhikari/A1283
	50	A2359	Ikuhikari/A1283
	51	KC260	Ikuhikari/A1289
	52	A2544	Ikuhikari/A1289
	53	A2543	Ikuhikari/A1289
	54	A2465	Ikuhikari/A1289//A1289
	55	A2468	Ikuhikari/A1289//A1289
	56	A2467	Ikuhikari/A1289//A1289
	57	A2473	Ikuhikari/A1289//A1289
	58	A2466	Ikuhikari/A1289//A1289
	59	A2472	Ikuhikari/A1289//A1289
	60	A2462	Ikuhikari/A1289//A1289
	61	A2478	Ikuhikari/A1289//A1289

Supplemental Table 1. (continued)

Year	Serial number	Line	Cross combination [a]
	62	A2471	Ikuhikari/A1289//A1289
	63	A2469	Ikuhikari/A1289//A1289
	64	A2475	Ikuhikari/A1289//A1289
	65	A2735	Akidawara/HC228
	66	A2734	Akidawara/HC228
	67	A2736	Akidawara/HC228
	68	A2263	Akidawara/A1323
2014	1	A2921	Satojiman/E233
	2	A2715	Satojiman/HC221
	3	N19	Satojiman/A1625
	4	N24	Satojiman/A1625
	5	A2782	Yamadawara*3/Satojiman
	6	A2856	Satojiman/E171/E227
	7	A2451	Satojiman//Ikuhikari/A1289
	8	N5	Satojiman//Ikuhikari/A1289
	9	N6	Satojiman//Ikuhikari/A1289
	10	N17	T71/Satojiman
	11	A2681	T71/Satojiman
	12	KC238	KC199/Satojiman
	13	KC244	Satojiman/E171
	14	KC256	Satojiman/T57
	15	N35	Satojiman/T65
	16	N36	Satojiman/T65
	17	N37	Satojiman/T65
	18	KC266	Satojiman/T65
	19	A2751	Satojiman/Tsuyahime
	20	N26	Satojiman/C129
	21	N27	Satojiman/C129
	22	N28	Satojiman/C129
	23	A2755	Satojiman/C129
	24	N29	Satojiman/C129
	25	A2757	Satojiman/C129
	26	N25	Satojiman/C129
	27	N38	Satojiman/C129
	28	N18	Satojiman/C129
	29	N23	Satojiman/A1628
	30	N33	Satojiman/A1628
	31	N34	Satojiman/A1628
	32	A2848	Satojiman/A1716
	33	A2851	Satojiman/A1853
	34	A2852	Satojiman/A1853
	35	A2861	Satojiman/E171//Tsuyahime
	36	A2862	Satojiman/E171//Tsuyahime
	37	A2863	Satojiman/E171//Tsuyahime
	38	A2864	Satojiman/E171//Tsuyahime
	39	A2865	Satojiman/E171//Tsuyahime
	40	A2866	Satojiman/E171//Tsuyahime
	41	A2911	KC245//Satojiman/I71
	42	A2601	KC247//Satojiman
	43	A2602	KC247//Satojiman
	44	N30	KC247//Satojiman
	45	A2604	KC247//Satojiman
	46	N31	KC247//Satojiman
	47	A2606	KC247//Satojiman
	48	N32	KC247//Satojiman
	49	A2904	Y70/Satojiman/A1882
	50	A2942	Y70/Satojiman//S139
	51	A2943	Y70/Satojiman//S139
	52	A2928	Genkitsukushi/Satojiman
	53	A2929	Genkitsukushi/Satojiman

Supplemental Table 1. (continued)

Year	Serial number	Line	Cross combination [a]
	54	A2930	Genkitsukushi/Satojiman
	55	A2931	Genkitsukushi/Satojiman
	56	A2932	Genkitsukushi/Satojiman
	57	A2935	Genkitsukushi/Satojiman
	58	A2936	Genkitsukushi/Satojiman
	59	A2937	Genkitsukushi/Satojiman
	60	KC251	Y70/Satojiman
	61	A2963	C132/Satojiman
	62	N7	Hinohikari/Satojiman
	63	KC260	Ikuhikari/A1289
	64	N3	Ikuhikari/A1289
	65	N2	Ikuhikari/A1289
	66	A2668	Ikuhikari/A1289//A1289
	67	A2669	Ikuhikari/A1289//A1289
	68	KC268	Ikuhikari/A1289//A1289
	69	KC269	Ikuhikari/A1289//A1289
	70	N11	Ikuhikari/A1289//A1289
	71	N8	Ikuhikari/A1289//A1289
	72	N9	Ikuhikari/A1289//A1289
	73	A2468	Ikuhikari/A1289//A1289
	74	N14	Ikuhikari/A1289//A1289
	75	N13	Ikuhikari/A1289//A1289
	76	N10	Ikuhikari/A1289//A1289
	77	A2473	Ikuhikari/A1289//A1289
	78	N12	Ikuhikari/A1289//A1289
	79	N15	N148/Ikuhikari
	80	N4	Akidawara/Ikuhikari
	81	N1	Akidawara/Hitomebore
	82	A2702	Akidawara*3/A1948
	83	N21	Akidawara/HC228
	84	N20	Akidawara/HC228
	85	N22	Akidawara/HC228
	86	A2691	CC200/Akidawara
	87	A2959	E235/Akidawara
	88	A2960	E235/Akidawara
	89	A2961	E235/Akidawara
	90	KC222	Akidawara/E176
	91	N16	KC166/Akidawara

[a] Yamadawara*3/Satojiman and Akidawara*3/A1948 indicate successively backcrossed three times to recurrent parents,Yamadawara and Akidawara, respectively.
